# Narcissistic and dependent traits and behavior in four archetypal 2-person, 2-choice games

**DOI:** 10.3389/fpsyt.2023.1275403

**Published:** 2024-01-08

**Authors:** Lawrence Ian Reed, Lily Cooke, Isabella Kasaba, Eleanor Harrison, Jill M. Hooley

**Affiliations:** ^1^Department of Psychology, New York University, New York, NY, United States; ^2^Department of Psychology, La Salle University, Philadelphia, PA, United States; ^3^Department of Psychology, Harvard University, Cambridge, MA, United States

**Keywords:** personality disorders, narcissistic personality disorder, dependent personality disorder, game theory, interpersonal

## Abstract

**Introduction:**

The characteristic behaviors we use to define personality pathology arise from specific interpersonal interactions. In an effort to create a laboratory-based context in which behavior might be expected to be influenced by particular personality traits, we used four 2-person, 2-choice games (the Prisoner’s Dilemma, Chicken, Leader, and Hero games) to create a simulated interaction and focused specifically on narcissism and dependency.

**Method:**

An online sample of 1137 (35% male, *M* age = 38.46 years, *SD* age = 13.20) participants completed brief, self-reported measures of trait narcissism and dependency and played one of the four games. Before deciding how to act or react, participants received either no message, a promise to cooperate, or a threat to defect from a (confederate) partner.

**Results:**

When receiving no message, those who cooperated in the Prisoner’s Dilemma had lower trait narcissism, while those who defected in the Chicken and Leader games had higher trait narcissism. Also with no message, participants who cooperated in the Hero game had higher trait dependency. Promises only affected the relationship between trait narcissism in the Leader game while threats only affected the relationship between trait dependency in the Chicken game.

**Discussion:**

These findings add to the limited behavioral research on personality pathology and largely support established interpersonal conceptualizations and models. Future work might extend these findings using even more ecologically valid approaches to explore the behavioral correlates of personality traits that have important implications for interpersonal interactions.

## 1 Introduction

### 1.1 The interpersonal perspective

Interpersonal dysfunction is a core feature of personality pathology that encompasses many of the peripheral features used to define the personality disorders. These include characteristic views of ourselves, others, and the world (1–3), motivations and emotions (4–7), as well as beliefs and attitudes (8). For example, a sense of entitlement (in narcissistic personality disorder) or a lack of remorse (in antisocial personality disorder), might motivate opportunistic behaviors that disregard the welfare of others. In contrast, the fear of losing a caretaker’s support (in dependent personality disorder) might motivate self-sacrificing behaviors that forgo one’s personal needs in order to keep others happy. As such, it has been argued that the most useful conceptualizations of personality disorders are interpersonal (9–13).

Traditional interpersonal perspectives propose that personality pathology is best understood as a stable pattern of thoughts, feelings, and motivations within the self that lead to characteristic behaviors when interacting with others (13). Furthermore, the interpersonal perspective assumes that the ultimate motivation behind these interpersonal processes and behaviors is an attempt to alleviate anxiety and maintain a positive self-image (13, 14). From this view, interpersonal behavior not only defines personality disorders, but also distinguishes them from other forms of psychopathology (15–17).

Contemporary integrative interpersonal theory [CIIT; (18–20)] retains the emphasis on the self in relation to others. Here, adaptive interpersonal functioning is defined by goal-oriented behavior that satisfies the needs of the self and another relative to developmental context and culture (20). In contrast, interpersonal dysfunction is defined as maladaptive social behavior resulting from a breakdown in regulating the self, emotions, or the interaction with another. It is argued that this dysfunction is a defining feature of personality pathology and can be captured in the *interpersonal situation,* which relates the self and another through perceptions and behaviors (13, 17, 20, 21).

The long tradition and continued influence of the interpersonal perspective is supported in the theoretical and empirical literature. Measures of psychosocial functioning significantly differ between those with and without personality disorders (22–25). This reinforces the idea that interpersonal dysfunction is a central component of personality pathology. The perspective has also formed a useful basis for understanding personality pathology. For example, empirically derived models such as the interpersonal situation (13, 17, 21) and interpersonal circumplex (14, 26) describe affects and motivations within the self in relation to another.

#### 1.1.1 Narcissism

Specifically, narcissistic personality disorder (NPD) is defined as a pervasive pattern of grandiosity, need for admiration, and lack of empathy (27). The diagnostic criteria for NPD capture the interpersonal dysfunction defined above. These include criteria directly related to the self in relation to another (grandiose sense of self-importance, belief that they are special and unique, requiring excessive admiration, a sense of entitlement, a lack of empathy, and an envy of others) as well as behaviors towards another (interpersonally exploitative and showing arrogant or haughty behaviors).

From the interpersonal perspective, those with NPD prioritize individual gains in status, esteem, and accomplishment at the expense of forming close, cooperative relationships with others (28–33). This overarching theme is consistent with self-reported data finding that those high in NPD rate themselves as more domineering (34), assertive (35), and antagonistic (35, 36). It is also consistent with interpersonal behaviors leaving those with NPD mired in conflict. For example, NPD is associated with problematic interpersonal behaviors such as violence (37) hostility (38) and aggression (39), often as a result of provocation from others. Consequently, those with NPD often have impaired social and occupational functioning (40) and significant distress (41).

#### 1.1.2 Dependency

Dependent personality disorder is defined as a pervasive and excessive need to be taken care of that leads to submissive and clinging behavior and fears of separation (27). As with NPD, many of the diagnostic criteria capture the interpersonal dysfunction described above. Criteria related to the self in relation to another include a need for reassurance from others, a need for others to assume responsibility for major areas in their life and feelings of discomfort and helplessness when alone. Criteria related to behaviors towards another include difficulty expressing disagreement, going to excessive lengths to obtain nurturance and support from others, discomfort when alone, and seeking another relationship when a close one ends. Taken together, the diagnostic criteria for DPD emphasize the inadequate and helpless features of the disorder (42).

These features create an interpersonal profile that contrasts with that of NPD. The characteristic beliefs of inadequacy and feelings of helpless motivate those with DPD to prioritize cooperation and harmony in interpersonal relationships. Those with DPD have been described by clinicians as conscientious and eager to please (43). Similarly, self-reported data from those with DPD suggest they are agreeable, amiable, and eager to acquiesce to others’ demands and expectations (44). Unfortunately, this comes at the expense of individual needs, which can result in interpersonal dysfunction (45–47). This includes difficulties with social and occupational functioning (43, 48) high risk sexual behavior (49) and domestic violence (42).

### 1.2 Integrating clinical science and game theory

Despite the emphasis on interpersonal dysfunction within the diagnostic criteria as well as within the theoretical and empirical literature, there have been few studies examining how those with personality disorders behave in social situations. Most work on personality pathology has utilized self-report measures, rating scales, checklists, clinical interviews, or projective techniques (50) in lieu of behavioral measures. Furthermore, there is relatively little research on dependent and narcissistic personality disorders relative to other personality disorders across all empirical works (51). In sum, the difficulty capturing social behavior in personality pathology and the relative lack of objective behavioral research methods has impeded the development of new knowledge in this area.

A recent advance in the study of personality pathology has come from the integration of game theory into clinical science. Economic games allow for the examination of interpersonal behavior in formalized social situations designed to capture those experienced in the real world. These situations are objective and easily compared across studies. Furthermore, they are not susceptible to the biases inherent in recollection, self-report, or clinical judgement.

The interpersonal situations that individuals with personality pathology encounter can be closely modeled using games. For example, individuals with narcissistic personality disorder might expect favorable treatment and exploit others. This might result in contentious behavior in competitive interactions such as the Prisoner’s Dilemma (52), Chicken (53, 54), or Auction (55). In contrast, individuals with dependent personality disorder might have difficulty expressing disagreement or assuming responsibility. This might result in passive or selfless behavior in cooperative games such as Hero or The Battle of the Sexes (52). Moreover, although these models represent simplified interactions, they may be used to predict behaviors in real life situations and provide primary treatment targets (56).

Interpersonal behavior in borderline personality disorder has been examined using the trust game (57). Here, participants must decide whether to invest in an investee. If the participant invests and the investee reciprocates, both can profit. However, if the participant invests and the investee does not reciprocate, the participant will suffer a loss. When playing this game once with another participant, those with BPD have been shown to invest less in the trustee in comparison to control participants (58). When played repeatedly, those with BPD were less likely to invest over repeated exchanges (59).

Narcissistic personality disorder has been examined using the commons dilemma (60). Here, participants must decide whether to take an action benefiting themselves at the cost of the group, or to benefit the group at the cost of the self. It has been found that trait narcissism, as measured by the Narcissistic Personality Inventory (NPI-40; (61)) was positively associated with behavior beneficial to the self at the cost of the group.

Narcissism has also been examined in the Prisoner’s Dilemma (52). The Prisoner’s Dilemma is a 2-person, 2-choice game similar to the commons dilemma. Here, participants must decide whether to cooperate or defect. The outcome of the game is dependent upon the decision that each player makes. If both players cooperate, they both receive a modest payoff. If they both defect, they both receive a smaller payoff. However, if one player cooperates and the other player defects, the cooperating player earns nothing while the defecting player earns the highest possible payoff. It has been found that criminal psychopaths from psychiatric hospitals were greater than 7 times more likely to defect in comparison to men chosen from the general population (62). Similarly, participants’ scores on a measure of psychopathy were positively associated with defection over time in a repeated Prisoner’s Dilemma game (63).

Finally, trait narcissism and dependency have been modeled using the Battle of the Sexes (52). Here, the interests of both players are shared, though not perfectly so. That is, participants share an interest in coordinating an outcome (either A or B). However, one player prefers to coordinate on Outcome A, whereas the other prefers they both coordinate on Outcome B. Results suggest that trait narcissism predicted behaving in accordance with one’s own interest while trait dependency predicted behaving in accordance with the other’s interests. Crucially, these effects were only found when participants were provoked with a message (either a promise to act in accordance with the other’s interests or a threat to act in accordance with one’s own interests) before making their decision (64).

The aim of the current investigation was to expand upon the empirical studies of interpersonal behavior in economic games. Here, we assessed participants’ trait narcissism and dependency and examined their behavioral decisions in four archetypal 2-person, 2-choice games: the one-shot Prisoner’s Dilemma (presented here as the Closed Bag Exchange) (52), Chicken (53, 54), Leader (65); and Hero (52) games. Based on previous literature suggesting that personality pathology is evoked in response to provocation (64, 66) each game was played in one of three communication conditions. In the first condition, participants received no message from their purported partner. In the second and third conditions, participants received either a preemptive promise of cooperation or threat of defection, respectively. Based on the interpersonal perspective of personality disorders as well as the extant empirical work, we hypothesized that participants who scored more highly in trait narcissism would be more likely to defect in each game whereas participants who scored more highly in trait dependency would be more likely to cooperate in each game.

This study was preregistered on the Open Science Framework^1^ and we report all measures, manipulations, data exclusions, and sample size determinations. De-identified data are available on OSF.^2^

## 2 Method

### 2.1 Participants

We recruited 1,260 participants through Prolific, which generates high quality data (67) and can provide good participant reimbursement. We planned to collect a sample size of approximately 100 per condition, and we did not analyze the data until all of the responses were collected. Assuming that cooperation and defection rates would be equal, this sample size provides 80% power to detect an effect size of *d* = 0.57 in a two-tailed test with a 5% false-positive rate (calculated with G*Power). One-hundred and twenty-three participants were excluded (120 failed at least one attention check and 3 revoked consent), leaving 1,137 participants’ (396 males, 738 females, 3 other). Participant’s mean age was 38.46 years (SD = 13.20) and their ethnicities were as follows: 75 Asian, 34 Black, 35 mixed, 968 White, and 25 other). Participants were currently residing in the United Kingdom (992) or in the United States (145). This study was approved by the New York University Institutional Review Board (IRB-FY2021-5214).

### 2.2 Measures

#### 2.2.1 Personality

We chose self-report measures of trait narcissism and dependency that were brief and applicable to both clinical and non-clinical samples. Trait narcissism was measured using the Narcissistic Personality Inventory [NPI-40; (61)]. In the NPI, participants are presented with 40 pairs of opposing statements and must choose the one that best describes them. For example, “I find it easy to manipulate people” (narcissistic response) and “I do not like it when I find myself manipulating other people” (non-narcissistic response). The NPI-40 assesses trait narcissism continuously on a range of non-clinical and clinical populations. Potential scores on the NPI range from 0 to 40. The NPI has shown good reliability and validity (68, 69). A recent meta-analysis has reported a mean population reliability coefficient of 0.82 (70).

Trait dependency was measured using the Dependent Personality Questionnaire (DPQ; 71). In the DPQ, participants are presented with eight questionnaire items and must rate the extent that it applies to them on a 4-point scale ranging from yes, definitely to no, not at all. Example items include “I am an independent person” and “I rely a lot on my family and friends.” Potential scores in the DPQ range from 0 to 24.

#### 2.2.2 Games

**Figure 1:**
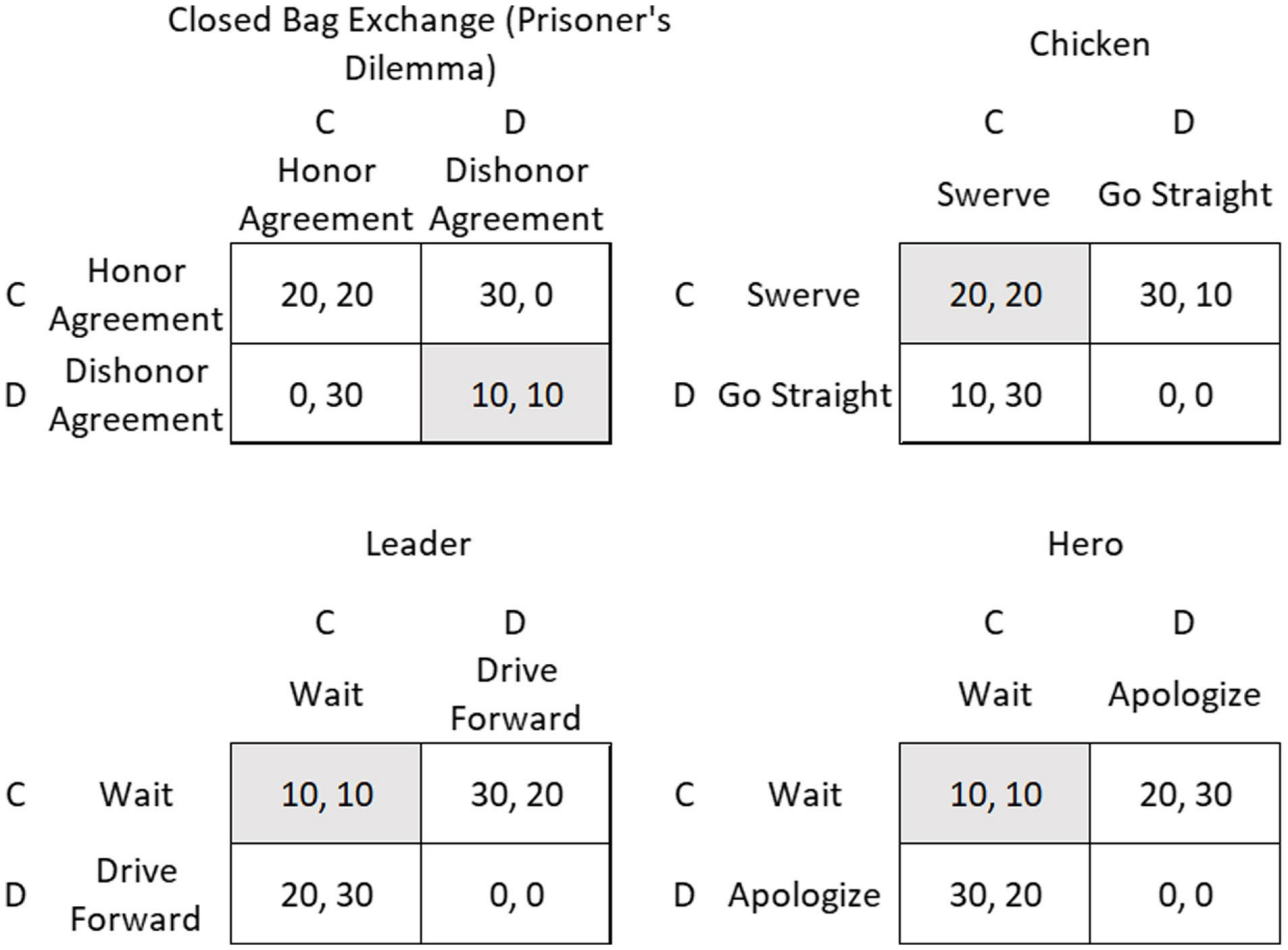
Game payoffs of four archetypal 2 × 2 games. Column payoffs presented first within each cell. Shaded cells are natural outcomes.

Participants played one of four symmetrical 2 -person, 2-choice games in which the outcome jointly depended upon each player’s independent decision to cooperate or defect (see Figure 1). As with previous works (72), we use the terms cooperate and defect throughout for convenience. They are not accurate in describing behavior in all the games. We chose the four 2 × 2 games described by Rapoport (65) as motivating distinct behaviors: the Prisoner’s Dilemma (*N* = 282), Chicken (*N* = 288), Leader (*N* = 279), and Hero (*N* = 288) games. Each game was defined by the relative rank of four ordinal payoffs (73) which have been referred in the literature as the Reward (R), Punishment (P), Temptation to defect (T), and the Sucker’s Payoff (S). Here, each payoff was set at either 0 cents, 10 cents, 20 cents, or 30 cents.

The payoff structure for each game is presented in Table 1. The decision that will minimize the player’s loss should they incur a loss is referred to as the maximin strategy. When each player chooses the maximin strategy, the results is the natural outcome represented in each payoff matrix. The motivation to switch from the maximin strategy is different in each game (see below).

**Table 1:** Payoff Structures for closed bag exchange (Prisoner’s Dilemma), chicken, leader, and hero games.

		Column	
		Cooperate	Defect
Row	Cooperate	(R)eward, (R)eward	(T)emptation to defect, (S)ucker’s payoff
	Defect	(S)ucker’s payoff, (T)emptation to defect	(P)unishment, (P)unishment

Column payoffs presented first within each cell. In Closed Bag Exchange (Prisoner’s Dilemma): T (30 cents) > R (20 cents) > P (10 cents) > S (0 cents). In Chicken: T (30 cents) > R (20 cents) > S (10 cents) > P (0 cents). In Leader: T (30 cents) > S (20 cents) > R (10 cents) > P (0 cents). In Hero: S (30 cents) > T (20 cents) > R (10 cents) > P (0 cents).

The order of payoffs in the Prisoner’s Dilemma is S > P > R > T. The Prisoner’s Dilemma is unique among the 4 games in that it has a dominant strategy (i.e., Nash equilibrium) in which each player will gain a greater individual payoff by switching from the minimax strategy regardless of their partner’s decision.

The Chicken, Leader, and Hero games also differ from the Prisoner’s Dilemma in that each player could benefit by switching from the minimax strategy if the other player does not. In the Chicken game (T > R > S > P), a player who switches (defects) benefits themselves at the expense of the other player. In the Leader game (T > S > R > P), a player who switches (defects) benefits themselves and the other player with the larger benefit being conferred upon the self. Finally, in the Hero game (S > T > R > P), a player who switches (cooperates) benefits themselves and the other player with the larger benefit being conferred upon the other player.

In a between-subjects design, each game was presented in one of three conditions varying in communication. In the no communication condition, participants did not receive any message from the other player. In the promise and threat conditions, participants received a message (that was presented as having come from the other player) communicating an intention to act cooperatively or selfishly, respectively. Participants were told that the message may or may not have reflected the other player’s actual decision.

### 2.3 Procedure

Participants gave consent and read a description of the procedure. Participants read that they would answer questions about their personality and play a game with another Prolific participant. The order that participants completed the personality measures personality measures and game was counterbalanced to reduce any potential order effects. Participants earned $1.93 for completing the task, and they could earn an additional bonus depending on the decisions made by them and the other participant. Most participants completed the task in under 10 min (*M* = 8.20, SD = 5.20).

A set of instructions was presented before each game. Participants read:

In this game, you will be matched with another Prolific participant. Although the story behind the game is fictional, the earnings are real. The decision that you make will affect your earnings in the game. Any money you earn in the game will be paid to you as a bonus payment.

These instructions were followed by a backstory and description of earnings unique to each game.

Closed Bag Exchange (Prisoner’s Dilemma).

The backstory was written as follows:

You and the other participant are meeting to exchange closed briefcases with the understanding that each of them contains valuable goods. Both you and the other participant can choose to either honor the agreement by putting the goods into the briefcase or dishonor the agreement by leaving the briefcase empty.

The earnings were described as follows:

If you and the other participant both honor the agreement, you both get the agreed upon goods and earn 20 cents.

If you and the other participant both choose to dishonor the agreement, you both keep your own goods without benefiting from the exchange and earn 10 cents.

If you choose to honor the agreement and the other participant chooses to dishonor the agreement, you end up with nothing and earn 0 cents.

If you choose to dishonor the agreement and the other participant chooses to honor the agreement, you end up with your goods and your partner’s goods and earn 30 cents.

In those conditions with a purported message, participants also read:

Before you decide, you’ll receive a brief, typical, written message from the other participant stating their choice. This message may or may not accurately reflect their actual decision.

After reading the instructions, participants answered 2 comprehension questions (e.g., “Let us say that the other participant decides to honor the agreement. How much money will you earn in bonus payments if you decide to dishonor the agreement?”). If they answered incorrectly, they could try again. Participants had to answer correctly in order to proceed.

Before making their decision, participants were shown a payoff matrix outlining the earnings described above. In those conditions with a purported message, participants then read “Here is a typical message from your partner.” This was followed by “I promise to honor the agreement and put the goods in the briefcase!” in the promise condition and “No way am I honoring the agreement! I’m leaving my briefcase empty!” in the threat condition. Participants then decided whether to honor or dishonor the agreement.

Chicken.

The backstory was written as follows:

You and the other participant are driving towards a single lane bridge from opposite directions. Both you and the other participant can choose either to go straight or to swerve away.

The earnings were described as follows:

If you and the other participant both swerve away, you will both show weakness and earn 20 cents each.

If you and the other participant both go straight, you crash and both earn 0 cents.

If you choose to swerve and the other participant chooses to go straight, you lose face and earn 10 cents.

If you choose to go straight and the other participant chooses to swerve, you show more strength and earn 30 cents.

In those versions with a purported message, participants also read:

Before you decide, you’ll receive a brief, typical, written message from the other participant stating their choice. This message may or may not accurately reflect their actual decision.

As in the Closed Bag Exchange, participants had to correctly answer 2 comprehension questions in order to proceed. Participants were shown a payoff matrix outlining the earnings described above. In those conditions with a purported message, participants then read “Here is a typical message from your partner.” This was followed by “I’m definitely going to swerve!” in the promise condition and “No way I’m swerving! I’m going straight!” in the threat condition. Finally, participants decided whether to swerve or go straight.

Leader.

The backstory was written as follows:

You and the other participant are driving in the same direction in separate lanes and must merge into a single lane. Both you and the other participant can choose to either wait or drive forward.

The earnings were described as follows:

If you and the other participant both wait, you lose time and earn 10 cents.

If you and the other participant both drive forward, you crash and earn 0 cents.

If you choose to wait and the other participant chooses to drive forward, your wait is minimal and you earn 20 cents.

If you choose to drive forward and the other participant chooses to wait, you move along quickly and you earn 30 cents.

In those versions with a purported message, participants also read:

Before you decide, you’ll receive a brief, typical, written message from the other participant stating their choice. This message may or may not accurately reflect their actual decision.

As in the other games, participants had to correctly answer 2 comprehension questions in order to proceed. Participants were shown a payoff matrix outlining the earnings described above. In those conditions with a purported message, participants then read “Here is a typical message from your partner.” This was followed by “I’ll wait!” in the promise condition and “I’m driving forward! Wait!” in the threat condition. Finally, participants decided whether to wait or drive forward.

Hero.

The backstory was written as follows:

You and the other participant are in an argument. Both you and the other participant can choose to either go to the other person’s house and apologize or wait at your house for an apology from the other participant.

The earnings were described as follows:

If you and the other participant both go to the other’s house to apologize, you both find an empty house and cannot apologize. The argument continues and you both earn 0 cents.

If you and the other participant both wait for the other to come to your house to apologizer, the argument continues. You both save face and earn 10 cents.

If you choose to go apologize and the other participants waits at their house for the apology, you lose face. But, the argument is resolved and you earn 20 cents.

If you choose to wait at your house for an apology and the other participant goes to your house to apologize, you save face. In addition, the argument is resolved and you earn 30 cents.

As in the other games, participants had to correctly answer 2 comprehension questions in order to proceed. Participants were shown a payoff matrix outlining the earnings described above. In those conditions with a purported message, participants then read “Here is a typical message from your partner.” This was followed by “I’m going to your house to apologize!” in the promise condition and “No way I’m going to apologize! I’m waiting at home for your apology!” in the threat condition. Finally, participants decided to go apologize or wait for an apology.

## 3 Results

### 3.1 Preliminary results

As preliminary analyses, we first examined cooperation rates in each game with no communication. To examine the effect of purported messages, we then compared cooperation rates with no communication to those in response to the purported promise and threat. In the Closed Bag Exchange, participants were more likely to cooperate (75/96, 78%) than defect (21/96, 22%) with no communication, Χ2 (1, *N* = 96) = 30.357, *p* < 0.01, ϕ = 0.563. In comparison to no communication, participants were no more likely to cooperate (72/93, 76%) than defect (21/93, 23%) in response to the promise, Χ2 (1, *N* = 189) = 0.014, *p* > 0.10, ϕ = 0.008, and less likely to cooperate (34/93, 37%) than defect (59/93, 57%) in response to the threat, Χ2 (1, *N* = 189) = 33.433, *p* < 0.01, ϕ = 0.421.

In the Chicken game, participants were more likely to cooperate (69/98, 70%) than defect (29/98, 30%) with no communication, Χ2 (1, *N* = 98) = 16.327, *p* < 0.01, ϕ = 0.408. In comparison to no communication, participants were no more likely to cooperate (59/94, 63%) than defect (35/94, 37%) in response to the promise, Χ2 (1, *N* = 192) = 1.261, *p* > 0.10, ϕ = 0.081, or in response to the threat, Χ2 (1, *N* = 192) = 0.304, *p* > 0.10, ϕ = 0.040.

In the Leader game, participants were more likely to cooperate (65/95, 68%) than defect (30/95, 32%) with no communication, Χ2 (1, *N* = 95) = 12.895, *p* < 0.01, ϕ = 0.368. In comparison to no communication, participants were less likely to cooperate (16/92, 17%) than defect (76/92, 83%), in response to the promise, Χ2 (1, *N* = 187) = 49.569, *p* > 0.01, ϕ = 0.515, and more likely to cooperate (84/92, 91%) than defect (8/92, 9%) in response to the threat, Χ2 (1, *N* = 187) = 15.115, *p* > 0.01, ϕ = 0.284.

In the Hero game, participants were less likely to cooperate (21/93, 23%) than defect (72/93, 77%) with no communication, Χ2 (1, *N* = 93) = 27.968, *p* < 0.01, ϕ = 0.548. In comparison to no communication, participants were less likely to cooperate (7/97, 7%) than defect (92/97, 95%), in response to the promise, Χ2 (1, *N* = 192) = 9.261, *p* < 0.01, ϕ = 0.220, and more likely to cooperate (56/96, 58%) than defect (40/96, 42%), in response to the threat, Χ2 (1, *N* = 189) = 25.010, *p* < 0.01, ϕ = 0.364.

### 3.2 Primary analyses

The primary analyses examining group differences in trait narcissism among participants who decided to cooperate and defect in each game are depicted in Table 2. In our sample, scores on the NPI ranged from 0–36 (*M* = 8.93, SD = 6.58) and scores on the DPQ ranged from 0–24 (*M* = 9.15, SD = 3.68). Consistent with our hypotheses, trait narcissism was higher among those choosing to defect in the Closed Bag Exchange, Chicken, and Leader games with no communication. These effects were in the moderate range (Cohen’s *d* = −0.469 -- -0.546). However, trait narcissism was no higher among those choosing to defect in the Hero game with no communication. Furthermore, trait narcissism was no higher among those choosing the selfish decision in all but one of the games with communication: the Leader game with the promise.

**Table 2:** Narcissistic personality inventory scores by game, communication, and decision.

	Decision								
	Cooperate			Defect			*t*(df)	*p*	Cohen’s *d*
	*N*	*M*	SD	*N*	*M*	SD			
Bag exchange									
None	75	7.800	6.049	21	11.333	7.825	−2.213 (94)	**0.015**	−0.546
Promise	72	7.806	5.616	21	10.110	6.743	−1.635 (91)	0.053	−0.405
Threat	34	7.353	5.933	59	8.848	6.337	−1.121 (91)	0.133	−0.241
Chicken									
None	69	8.464	6.723	29	11.966	6.483	−2.378 (96)	**0.010**	−0.526
Promise	59	8.864	6.418	35	9.029	5.592	−0.126 (94)	0.450	−0.027
Threat	71	8.113	6.767	25	9.000	7.165	−0.555 (94)	0.290	−0.129
Leader									
None	65	6.892	5.593	30	9.633	6.365	−2.125 (93)	**0.018**	−0.469
Promise	16	6.438	4.746	76	10.224	6.732	−2.136 (90)	**0.018**	−0.588
Threat	84	8.381	7.021	8	10.375	5.927	−0.776 (90)	0.220	−0.287
Hero									
None	72	10.083	6.560	21	8.143	8.696	1.104 (91)	0.136	0.274
Promise	92	9.826	6.519	7	13.143	8.092	−1.276 (97)	0.102	−0.500
Threat	40	9.450	6.976	56	9.607	7.274	−0.106 (94)	0.458	−0.022

Bolded values, *p* < .05.

The primary analyses examining group differences in trait dependency among participants who decided to cooperate and defect in each game are depicted in Table 3. Contrary to our hypotheses, trait dependency was no higher among those choosing the selfish decision in the Bag Exchange, Chicken, or Hero games with no communication. Consistent with our hypothesis, trait dependency was higher among participants who decided to cooperate in the Hero game with no communication. This effect was near the moderate range (Cohen’s *d* = 0.468). Finally, trait dependency was no higher among those choosing the cooperative decision in all but one of the games with communication: the Chicken game with the threat.

**Table 3:** Dependent personality disorder questionnaire scores by game, communication, and decision.

	Decision								
	Cooperate			Defect			*t*(df)	*p*	Cohen’s *d*
	*N*	*M*	SD	*N*	*M*	SD			
Bag exchange									
None	75	9.453	4.004	21	9.048	4.727	0.394 (94)	0.347	0.097
Promise	72	8.639	3.735	21	9.000	3.738	−0.390 (91)	0.349	−0.097
Threat	34	9.824	3.424	59	8.661	3.726	1.492 (91)	0.070	0.321
Chicken									
None	69	9.073	2.957	29	8.690	4.676	0.488 (96)	0.313	0.108
Promise	59	10.000	3.333	35	9.114	3.113	1.276 (92)	0.103	0.272
Threat	71	9.789	3.153	25	8.200	3.440	2.116 (94)	**0.018**	0.492
Leader									
None	65	9.554	3.531	30	8.700	3.131	1.134 (93)	0.130	0.250
Promise	16	10.000	4.442	76	8.724	3.268	1.329 (90)	0.094	0.366
Threat	84	9.179	3.638	8	7.125	4.257	1.504 (90)	0.068	0.557
Hero									
None	21	11.143	4.199	72	9.264	3.961	1.887 (91)	**0.031**	0.468
Promise	7	8.000	3.651	92	8.696	3.560	−0.498 (97)	0.310	−0.195
Threat	56	9.464	4.242	40	8.425	4.038	1.207 (94)	0.115	0.250

Bolded values, *p* < .05.

## 4 Discussion

We examined participants’ behavior in four 2-person, 2-choice games in relation to trait narcissism and dependency in one of three preemptive message conditions. We hypothesized that participants with high trait narcissism would be more likely to defect while participants with high trait dependency would be more likely to cooperate in each game across conditions. Consistent with our hypotheses, trait narcissism was higher among participants who defected in the Closed Bag Exchange (Prisoner’s Dilemma), Chicken, and Leader games in comparison to those who cooperated when given no preemptive message. However, trait narcissism did not differ by behavior in the Hero game with no preemptive message. In response to preemptive messages, trait narcissism was no higher among participants who defected across games with one exception: those who defected in the Leader game reported higher trait narcissism compared to those who cooperated in response to a promise of cooperation.

These results are consistent with interpersonal conceptualizations emphasizing behaviors that prioritize the self over the other (13, 14, 74). Results from the no communication condition are also consistent with previous studies showing that psychopaths are more likely to defect in Commons (60) and Prisoner’s Dilemma (62, 63) games. They are, however, only partially consistent with previous research examining trait narcissism and dependency in the Battle of the Sexes (BoS) game. This research found that trait narcissism predicted participant defection in the BoS game only when participants were provoked with a preemptive threat from their partner (64). These results might be reconciled when considering participants’ partners. Here, participants were told they were playing with another Prolific participant while Simon and Reed (64) told participants to imagine they were playing with someone who was very close to them.

Also consistent with our hypotheses, trait dependency was higher among participants who cooperated in the Hero game in comparison to those who defected when given no preemptive message. However, trait dependency was no higher among participants who cooperated in the Closed Bag Exchange (Prisoner’s Dilemma), Chicken, or Leader games in comparison to those who defected when given no preemptive message. In response to preemptive messages, trait dependency was no higher among those who cooperated with one exception: those who cooperated in the Chicken game reported higher trait dependency compared to those who defected in response to the threat.

These results add nuance to interpersonal conceptualizations positing that individuals with dependent personality disorder behave submissively (43, 75, 76). They are also partially consistent with previous research. Simon and Reed (64) found that trait dependency predicted participant cooperation only when participants were provoked with a preemptive threat from their partner. Here, we found the opposite: trait dependency predicted cooperation only in the no communication condition. As with trait narcissism in the BoS game, these differences could be the result of partners’ portrayal (77, 78).

Clinically, these behaviors can be used to model those behaviors characteristic of individuals with trait narcissism and dependency in real life situations. For example, the selfish behaviors seen here among those high in trait narcissism in the Closed Bag Exchange (Prisoner’s Dilemma) game may relate to dysfunctional behavior in intimate relationships (79). In contrast, the selfless behaviors seen here among those high in trait dependency may related to the increased risk for physical abuse from spouses (80). Our findings also support the value of adopting a more dimensional and trait-focused approach to personality (81, 82) – an approach that is now receiving considerable theoretical attention (83, 84).

Our results should be interpreted within the context of the methodology and sample. The payoff structure inherent in each game created a unique interpersonal situation for participants to behave in a range of ways. This could account, in part, for varying behaviors among individuals with similar personality pathology across different games. Furthermore, as shown in the preliminary analyses, preemptive promises and/or threats affected participants’ behavior in every game aside from Chicken. Additionally, factors such as group identity, anonymity, repetition, and the relative magnitude of game payoffs have all been shown to affect behavior (85–87). Furthermore, our online sample was a non-clinical population consisting mostly of females. Females have been shown to differ from males in the prevalence and presentation of NPD ((88–90) and DPD (88). We also do not know if our participants would have behaved differently had they interacted with their partners in person. That said, it is possible that not seeing their interactional partner allowed participants to behave in a more authentic manner than might otherwise have been the case. It also warrants mention that many online interactions are characterized by anonymity and a lack of face-to-face interaction. As such our findings may have more ecological validity than might be apparent at first glance. Finally, an updated validation of the NPI-40 might further contextualize the validity and reliability of our data.

In sum, interpersonal behavior in economic games can be used to test interpersonal conceptualizations of personality pathology. Here, we found that those behaving selfishly in the Chicken and Leader games reported higher trait narcissism while those behaving cooperatively in the Prisoner’s Dilemma game reported lower trait narcissism in instances without communication. This suggests a narcissistic preference for self-interested behavior regardless of its effect on the other. In addition, we found that those behaving cooperatively in the Hero game reported higher trait dependency in instances without communication. This suggests a dependent preference to sacrifice a benefit in order to help another. Further research utilizing similar methods stands to increase our understanding of personality pathology across social situations and levels of communication.

## Data availability statement

Publicly available datasets were analyzed in this study. The de-identified data can be found on the Open Science Framework website: https://osf.io/xturq/?view_only=e193967749354534b9c06cfc587ddbc3; https://osf.io/hxzse/?view_only=82f73da95df549bf95822181b7b05df5. Further inquiries can be directed to the corresponding author.

## Ethics statement

The studies involving humans were approved by New York University Institutional Review Board. The studies were conducted in accordance with the local legislation and institutional requirements. The participants provided their written informed consent to participate in this study.

## Author contributions

LR: Conceptualization, Data curation, Formal analysis, Investigation, Methodology, Writing – original draft, Writing – review & editing. LC: Writing – review & editing. IK: Writing – review & editing. EH: Writing – review & editing. JH: Conceptualization, Supervision, Writing – review & editing.

## Footnotes

^1^https://osf.io/xturq/?view_only=e193967749354534b9c06cfc587ddbc3

^2^https://osf.io/hxzse/?view_only=82f73da95df549bf95822181b7b05df5
